# The Surgical Significance of Vomiting

**Published:** 1908-06-06

**Authors:** 


### THE SURGICAL SIGNIFICANCE OF VOMITING.

### IV.—ITS DIAGNOSTIC VALUE (*continued*).

It is often important in a case in which the diagnosis lies between gastric and duodenal ulcer to be able to differentiate between the two as far as one can from the history; and this is especially true if symptoms pointing to the perforation of an ulcer have already made their appearance when one is first called to the case. If the case can be more or less clearly shown to be one of gastric ulcer the abdomen may be opened in the middle line of the epigastrium, but if the history points to a duodenal ulcer a more satisfactory access is gained by a vertical incision through the upper part of the right rectus abdominis muscle. In doubtful cases the latter incision should also be chosen, for the reason that a gastric ulcer is more often situated at the pyloric than towards the cardiac end of the stomach, and generally upon the anterior wall. Of all gastric ulcers the great majority are situated upon the posterior wall, but, fortunately for the surgeon, of all those that perforate by far the greater number are situated upon the anterior wall. The exact reason for this is not very clear. It may be that there is a greater range of movement in the anterior stomach wall, which prevents the formation of adhesions, and so leads to peritonitis rather than to abscess formation.

Can we, therefore, distinguish between gastric and duodenal ulcers from the symptoms alone? The character of the vomiting may help us. In a gastric ulcer it occurs from one to two hours after the ingestion of food. In a duodenal ulcer it may be deferred as much as three or four hours. This is, after all, what would be expected. It is only when the stomach contents have passed through the pylorus, a process which takes some time, that the duodenal ulcer is irritated, and this is the immediate cause of the vomiting. Hæmatemesis, as such, is more common in the gastric form: in duodenal ulcer melæna is the more usual manifestation of loss of blood from the surface of the ulcer. Again, in gastric ulcer the patient experiences pain and tenderness in the left half of the epigastrium, whereas in a typical duodenal ulcer these symptoms are both found upon the right side.

But to show that these divergences are not an absolute guide to the differential diagnosis of the two conditions, two cases which came under observation within a day or two of one another may be briefly quoted. Both had, when first seen, obvious peritonitis starting in the upper abdominal segment with a history of the sudden onset of severe abdominal pain pointing to a perforative lesion. In both laparotomy was performed and both were found to be suffering from a perforated duodenal ulcer. In the first case the history was typical. The patient, a man, had had indigestion and vomiting after food for some months. The vomiting occurred from three to four hours after meals, and was accompanied by pain in the right half of the epigastrium. He had hæmatemesis, but no melæna. In the second case the history, however, was quite indefinite. The patient, also a man, had had slight attacks of indigestion on and off for years, but no vomiting nor hæmatemesis. He had sometimes had slight diffuse pain in the epigastrium; and yet this patient had a perforation of a duodenal ulcer which was not an acute one, but must have existed for at least several months.

In all cases in which vomiting is a symptom much may be learnt by an examination of the vomited material, or the same result may be obtained by examining what is called a test meal which has been introduced into the stomach and withdrawn by means of a rubber tube. A test meal as usually given consists of about 100 grams of bread or toast and from three to four hundred cubic centimetres of plain water or tea made without milk. It is better to wash out the stomach previously. The fluid removed is examined chemically and microscopically.

The most important thing to test for is the presence of free hydrochloric acid. The fact that litmus is turned red by the fluid is not positive proof of this, since litmus will react to the presence of acids in combination. A fairly delicate test, and one which is easy of application, is to mix with the solution some tropæolin, which turns pink in the presence of free hydrochloric acid. The absence of free hydrochloric acid is suggestive of the presence of carcinoma; and a very interesting observation has recently been made that the hydrochloric acid in the stomach is diminished or even entirely absent not only if there is carcinoma of that organ, but even if the patient is suffering from carcinoma in some quite remote part of the body. Time has yet to show whether the chemical change in the cells implied by the absence of free hydrochloric acid has any causal relationship to malignant disease.

If free hydrochloric acid is absent, lactic acid will probably be found. The two are not found together. Lactic acid may be tested for by the addition of Uffelmann's reagent. This consists of a solution of ferric chloride in weak carbolic acid. It is normally a light purple colour, but in the presence of lactic acid it turns yellow.

All these phenomena point to the existence of a carcinoma, but no evidence can be regarded as positive, short of finding cells in the material which present the appearance of carcinoma under the microscope. The converse, however, is not true; that is, the presence of normal cells from the gastric mucous membrane is not proof that carcinoma is absent. As far as the presence of blood is concerned, more may be learnt clinically from an inspection of the vomit than from a microscopical examination of a test meal. Thus the vomiting of large quantities of blood at infrequent intervals points to gastric ulcer, while the constant appearance of small amounts of blood in the vomit is much more suggestive of carcinoma. In either case microscopical evidence of the presence of altered blood may be found in the shape of corpuscles or pigment, but such a discovery is of no value in establishing a differential diagnosis.

Thus, the examination of a test meal may help the diagnosis, but it rarely makes it positive. It may happen, for instance, that if carcinoma has been preceded by a gastric ulcer, the hydrochloric acid may still be in excess instead of deficient.

